# Extracted Triterpenes from *Antrodia cinnamomea* Reduce the Inflammation to Promote the Wound Healing *via* the STZ Inducing Hyperglycemia-Diabetes Mice Model

**DOI:** 10.3389/fphar.2016.00154

**Published:** 2016-06-13

**Authors:** Yu-Sheng Wu, Shiu-Nan Chen

**Affiliations:** College of Life Science, National Taiwan UniversityTaipei, Taiwan

**Keywords:** triterpenes, hyperglycemia, antiinflammatory activity, STZ-induced diabetic mice, wound healing

## Abstract

This research evaluated the effects of triterpenes on the regulation of STZ-induced hyperglycaemic diabetes through an anti-inflammatory response. Diabetic mice were orally administered various concentrations of triterpenes on a daily basis. Weight gain, volume of drinking water, and liver and spleen weight were recorded and evaluated. These evaluations presented a positive regulation to the abnormal metabolism appearance compared to the diabetic mice. In the diabetic mice, the detection of adiponectin production or elevated levels of inflammatory factors such as CCL1 and TPO expression were found to reduce hyperglycaemia and thereby induce an inflammatory response. Moreover, to the best of our knowledge, hyperglycaemia impairs the tissue healing associated with an increased and prolonged inflammatory response. An investigation of the anti-inflammatory response in wound healing as affected by the triterpenes verified the promotion of wound recovery.

## Introduction

Diabetes is a metabolic disease characterized by hyperglycaemia resulting from defects in insulin secretion or function, and is associated with the long-term damage, dysfunction, and failure of various organs, especially the eyes, kidneys, nerves, heart, and blood vessels (Mellitus, 2005). Kidney and liver dysfunction are associated with diabetes complications in rodents. Various studies have indicated that diets high in saturated fat and cholesterol contribute to hypercholesterolemia and metabolic disturbances, which may cause hyperglycaemic diabetes in humans and animals (Mathé, 1995; Kuller, 2006; Harris et al., 2009). Hyperglycaemia can rapidly become severe hyperglycaemia and/or ketoacidosis in the presence of infection or other stress (Mellitus, 2005). The inducing stress can result from the presence of excessive counterregulatory hormones (glucagon, growth hormone, catecholamine, and glucocorticoid; either endogenous or exogenous) and high circulating or tissue levels of inflammatory cytokine (McCowen et al., 2001). Diabetes mellitus has been postulated as representing an innate immune system disease responsible for an inflammatory cytokine-mediated acute phase response (Pickup et al., 1997). Furthermore, hyperglycaemia-induced oxidative stress, with soluble advanced glycation end products (AGE) and products of lipid peroxidation, has been reported as possibly being a key activator of upstream kinases, thereby inducing inflammatory gene expression. Moreover, intake of glutathione (an antioxidant) can completely prevent the increase of inflammatory cytokines (Giugliano et al., 1996; Schmidt et al., 1999).

*Antrodia cinnamomea* is a popular medicinal mushroom reported to possess antitumor and immunomodulating activities with anti-inflammatory effects (Cheng et al., 2005). *A. cinnamomea* has significant apoptotic effects against leukemia HL-60 cells, suggesting that the species may possess protective antioxidants and anticancer properties (Hseu et al., 2004). Recently, researchers have studied the biological effects of bioactive components extracted or purified from its fruiting bodies, namely pure culture mycelia and culture filtrate. Furthermore, bioactive components have been isolated from *A. cinnamomea*, including triterpenoids, polysaccharides, proteins, fatty acids, and phenyl derivatives (Shen et al., 2004; Ao et al., 2009; Yu et al., 2009). Notably, triterpenoids, a large and structurally diverse group of natural products derived from squalene or related acyclic 30-carbon precursors, are uniquely abundant with well-characterized biological activities of modulation on the immune cells (Minns et al., 2004).

According to these findings and hypotheses, we used triterpenes extracted from a subculture of *A. cinnamomea* to examine anti-inflammatory responses in STZ-induced hyperglycaemic mice. The blood glucose level, drinking water volume, liver and spleen weight, and TPO, CCL1, and adiponectin concentrations of the mice were recorded. Furthermore, we used triterpenes with high anti-inflammatory properties to observe the effect of triterpenes on wound healing in the mice.

## Materials and methods

### Subculture of *Antrodia cinnamomea*

The *Antrodia cinnamomea* BCRC 36401 was purchased from the Bioresources Collection and Research Center (BCRC), Food Industry Research and Development Institute, Hsinchu, Taiwan. After transferring to the laboratory, the *Antrodia cinnamomea* was subculture into the oats containing with 5% glucose (16301, RDH) and 1% yeast extract (09182, SIGMA) within the vent plug-glass bottles at 22°C with a 12 h light for 30 days. After the mycelium was observed to overlap on the cultured oats, the mycelia were separated from fermented broth and washed with distilled water. Finally, the mycelia were freeze-dried to powders. The freeze-dried powder was initiated into the 80°C hot water for 6 h (powder: hot water = 1: 100) to separate the water-soluble materials. After remove of water-soluble materials, the extraction of triterpenes was using 99.8% water-free ethanol (SIGMA) (the removed water-soluble powder: water-free ethanol = 1: 50) in 1.5 h for 3 times and the extracted compound was following to freeze-dried as the investigation sample and analyzation of triterpenes species by HPLC.

The analyzation of triterpenes was using of HPLC and the analyzation condition was presented as:

**Table:** 

**Sample injection volume**	**Flow rate**	**Detection wave length**	**Column**	**Running liquid (A)**	**Running liquid (B)**
2 μl	200 μl/min	244 nm	C18	99.99% H_2_O + 0.01% H_3_PO_4_	99.99% ACN + 0.01% H_3_PO_4_

### Animals

All study procedures were performed in accordance with protocol approved by the National Taiwan University Animal and Use Committee (NTUAUC). We used 120 of 6 weeks old male ICR mice (*N* = 5, purchased from Laboratory Animal Center, National Taiwan University College of Medicine) for the diabetes physiological observation experiment and 25 of 6 weeks old male ICR mic (*N* = 5, purchased from Laboratory Animal Center, National Taiwan University College of Medicine) were examined in the wound healing. For the present study, animals were housed in the Animal Housing Facility of National Taiwan University, College of Life Science using polycarbonate cages with paddy husk bedding in the animal room. The room temperature and relative humidity were maintained at 21 ± 2°C and 55 ± 20%, respectively, with a 12 h light/dark cycle.

### Induction of diabetes

The inducing of diabetes ICR procedure was followed with the presented protocol (Dekel et al., 2009). Briefly, to induce diabetes in mice, administration of a single *i.p*. injection of 180 mg STZ (Sigma Chemical Co., St. Louis, MO) per kg mouse body weight. STZ was freshly dissolved in cold citrate buffer (0.05 M; pH 4.5). Mice were fasted for 20 h before STZ injection. The each experimental mouse was weighed before injection with STZ and continued to weigh mice daily until the end of the experiment. The blood of each mouse was obtained from the tail vein and measurement of blood glucose by using glucose meter (Roche Accu- Chek Performa). The mice with blood glucose above 235 mg/dL were considered to be diabetic and used for the experiment.

### Experimental protocol

The mice were divided into 6 groups each comprising with 25 indivisal experimental diabetes or not diabetes (control) mice. Extracted triterpenes were dissolved in 5% DMSO and was diluted in water following to daily oral with 200 μL volume of various concentration or glybenclamide (600 μg/kg b.w.) (SIGMA) which is as a positive control for 35 days. The body weight of each mouse from various groups were recorded in the scarified day. In the sacrificed day, five of selected experimental mice were fasted overnight, and sacrificed by over dose injection of Ketamine (60 mg/kg b.w. intramuscular injection).

### Serum analysis

Concentrations of serum thrombopoietin (TPO) (EK0517, Boster), CCL1 (EK0566, Boster) and adiponectin (EK0596, Boster) were measured by the ELISA detection system. Briefly, separate 96-well plates were pre-coated with the anti-mouse TPO, CCL1 or adiponectin antibody. One hundred microliters of serum or standard were added, and the plates were incubated at room temperature. Plates were washed for 3 times and the secondary antibody was added and incubated at room temperature for 1 h. After incubation, plates were then washed 5 times and the substrate solution was added and to be incubated in the dark. The reaction was stopped by the stop solution and the absorbance was read at 450 nm by Microplate Reader.

### Diabetes mouse skin wound healing examination

The experiment procedure was following the wound healing model assay (Gal et al., 2008; Yang et al., 2011; Hsiao et al., 2015). All study procedures were performed in accordance with protocol approved by the National Taiwan University Animal and Use Committee (NTUAUC). We used 15 of 6 weeks old male ICR (*N* = 3, purchased from Laboratory Animal Center, National Taiwan University College of Medicine) for this experiment. For the present study, animals were housed in the Animal Housing Facility of National Taiwan University, College of Life Science using polycarbonate cages with paddy husk bedding in the animal room. The room temperature and relative humidity were maintained at 21 ± 2°C and 55 ± 20%, respectively, with a 12 h light/dark cycle. One of 1.5 × 0.5 cm full thickness incision wound was made in one of the regions. Each wound was cleaned by the 3M Cavilon™ No Rinse-Skine Cleanser then to spray with 3M Cavilon™ No Sting Barrier Film solution with or without investigation sample. The treatment triterpenes were pre-dissolved into 5% DMSO and diluted into 3M Cavilon™ No Sting Barrier Film solution. The treatments were stored at into a spray bottle and using to spray with 700 μL on the wound surface interval 3 days to be observed the wound healing. The experiment group was divided into four groups included of 1. Control group: sprayed of 3M Cavilon™ No Sting Barrier Film, 2. Diabetes: sprayed of 3M Cavilon™ No Sting Barrier Film, 3. Diabetes mice sprayed of 5 mg/kg triterpenes with 3M Cavilon™ No Sting Barrier Film, 4. Diabetes mice sprayed with 10 mg/kg triterpenes with 3M Cavilon™ No Sting Barrier Film, and 5. Diabetes mice sprayed with 20 mg/kg triterpenes with 3M Cavilon™ No Sting Barrier Film.

### Statistical analysis

The experimental data in each treatment group were divided by the control group. Scheffe's and two-way ANOVA were used to analyze the statistical significance between treatment and control groups. A *p*-value less than 0.05 as marked as^*^ and less than 0.01 as marked as^**^ was considered to be statistically significant. Results were presented as means ± SEM.

## Results

### Triterpenes analysis

Investigation sample were prepared by ethanol extraction method. In the initiation, the water-soluble materials of freeze-dried mycelium powder were removed by hot water extraction, after remove, the powder was in the process of ethanol extraction to collect the triterpenes for investigation and analysis.

**Figure 1:**
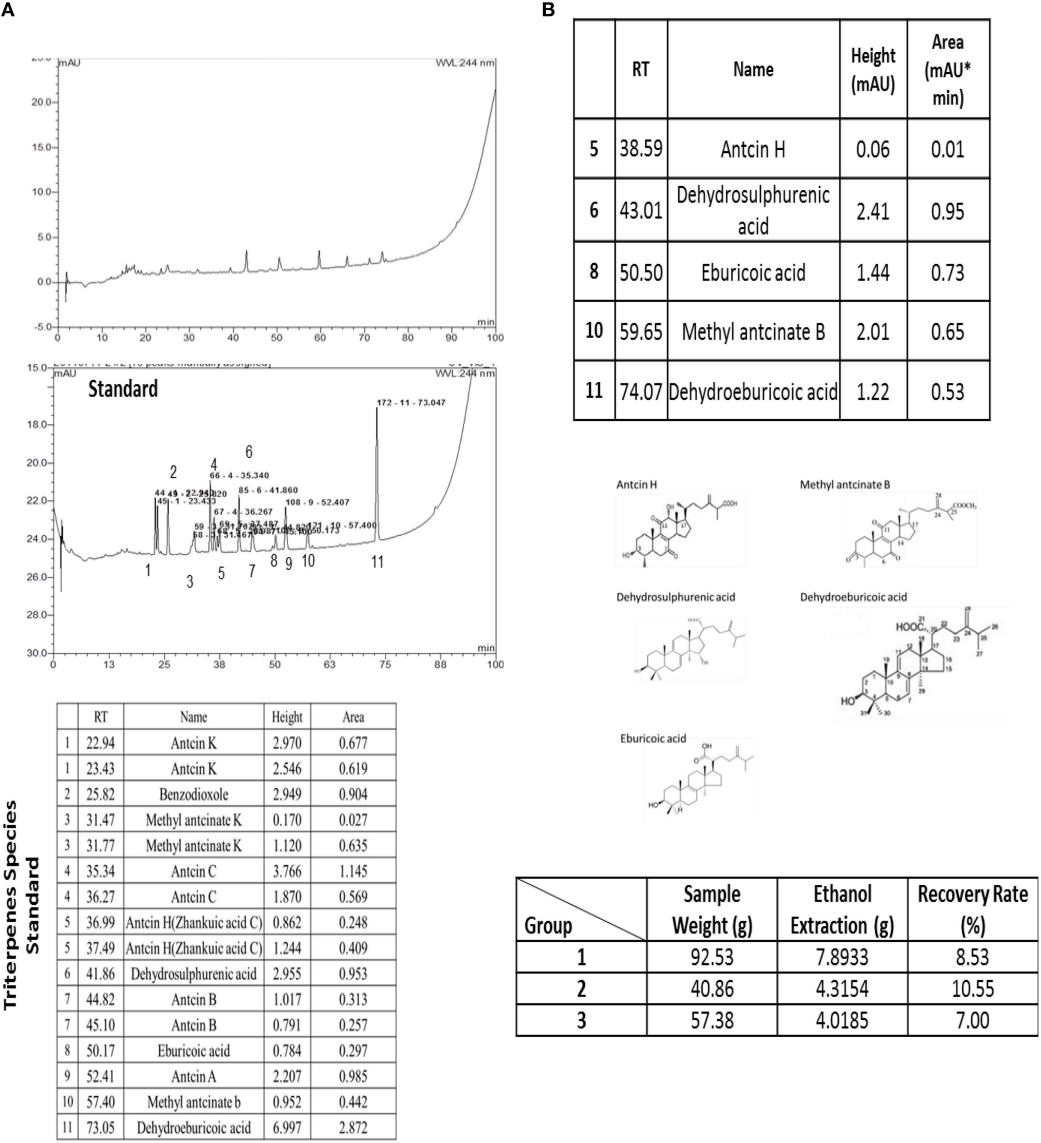
**The extracted triterpenes was analyzed to identify the species of triterpenes by using of the HPLC (A)**. The extracted triterpenes was major included of Antcin H, Dehydrosulphurenic acid, Eburicoic acid, Methyl antcinate B and Dehydroeburicoic acid **(B)**. Recovery rate of the extracted triterpenes was recorded with three repeats.

The species analysis result was presented as Figure 1A and the triterpenes recovery was measured as presented as Figure 1B. The extracted material was comparison to the 11 species of triterpenes and the results was indicated that it was including of Antcin H, Dehydrosulphurenic acid, Eburicoic acid, Methyl antcinate B and Dehydroeburicoic acid analyzing by the HPLC. The recovery rate was measured from different weight of the powder and the presented recovery rate was between 7 and 10% by three times.

### Physiological observation

**Figure 2:**
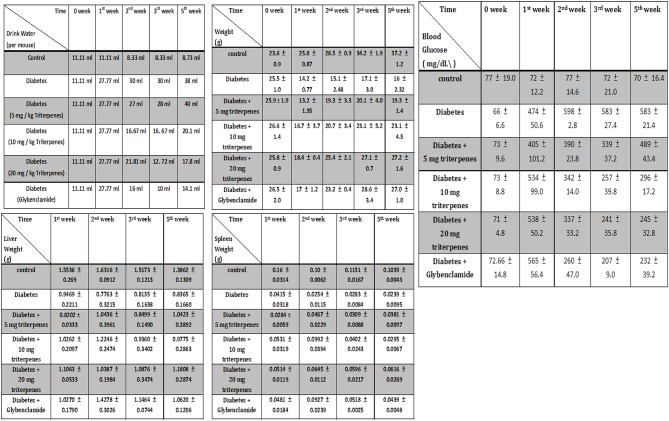
**The appearance of drinking water volume, weight, liver weight, spleen weight and the blood glucose was weekly measured and recorded**. (*n* = 5, Results were presented as means ± SEM).

**Figure 3:**
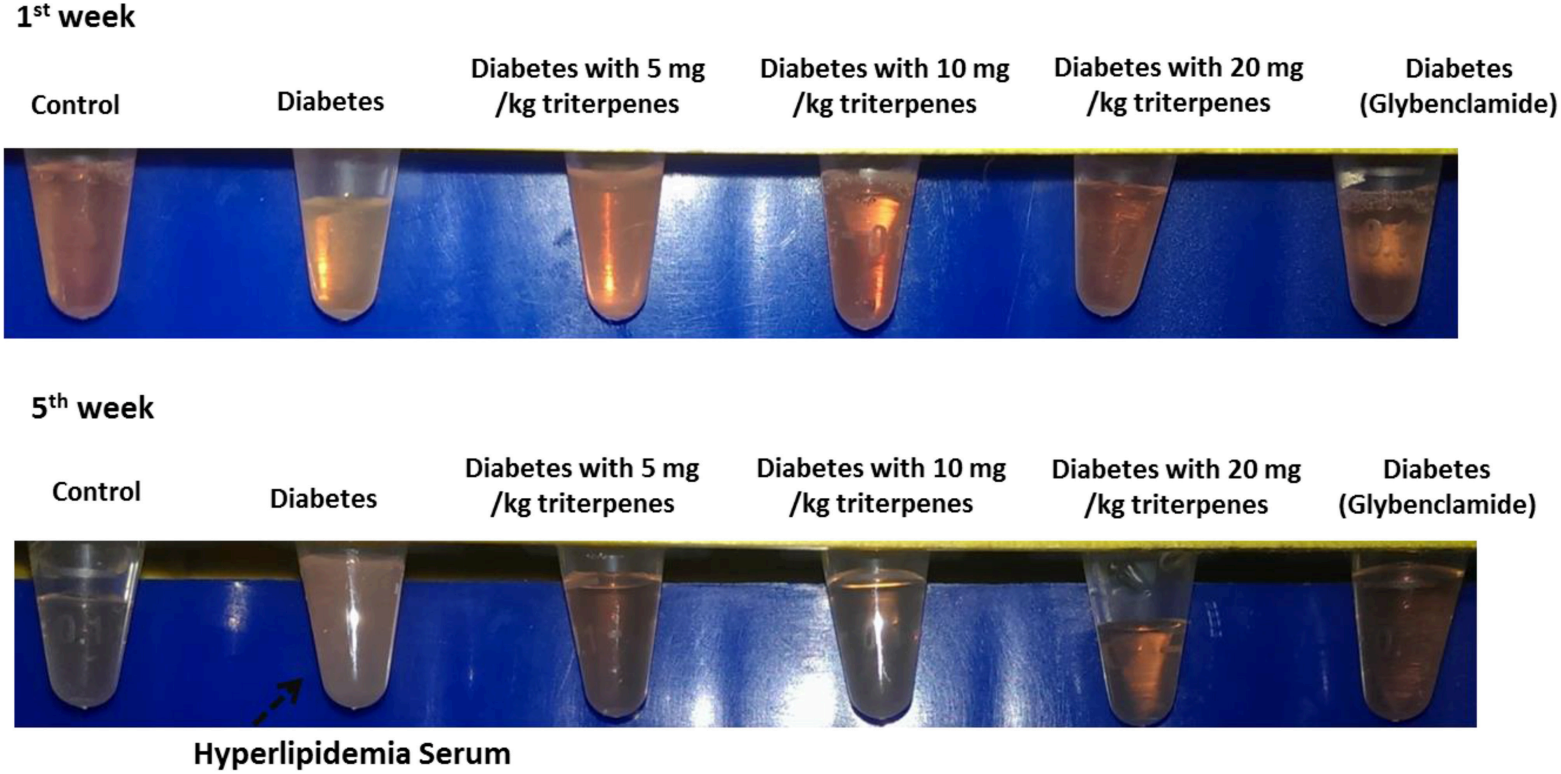
**The observation of serum and it was presented as the hyperlipidemia seurm in the 5th week**.

Investigation of daily intakes of triterpenes, the body weight gain of diabetic mice feeding with investigation triterpenes and control mice were significantly higher (*p* < 0.05) than the diabetes mice as presented in Figure 2 and the drinking water volume was observed and recorded every week to present as drinking water (ml)/per mouse. Observation of the drinking water, the treatment groups and control were shown to be lower than the diabetes group (*p* < 0.05) (Figure 2). Measurement of the liver and spleen weight in the sacrificed mice, the average of the control mouse liver weight was presented as 1.3862 ± 0.1309 g and spleen weight was presented as 0.1039 ± 0.0043 g in the end of the experiment. However, the liver weight of diabetes group was presented as 0.8365 ± 0.1660 g as lower than the control and treatments of triterpenes (*p* < 0.05) that is also similar in the spleen weight observation. The blood glucose of the control was presented as the average of 70 ± 16.4 mg/dL and the diabetes group was presented as higher concentration of 583 ± 21.4 mg/dL than the treatment groups and control in the weekly observation (*p* < 0.05). Interestingly, the observation of the serum in the various groups and it was presented the hyperlipidemia serum in the diabetes group but not presented in the other group in the end of the investigation (5th week) (Figure 3). The hyperlipidemia serum was presented as haze even filtrated by the 0.22 μm filter but the other groups was presented as clarifying. The appearance observation was shown that the diabetes group mice were loosen in the hair especial in the nose and oral area presented as old mouse but not presented in the other treatment groups (Figure 3).

### Serum biological analysis

Diabetes caused an increase in circulating thrombopoietin (TPO), in the observation of TPO expression analyzing the ELISA measurement, it was found that daily oral with various concentration of triterpenes was exactly able to reduce the concentration of serum TPO from circulation system. The presented data was pointed to reduce TPO concentration especially in the oral with 10 mg/bw-kg, 20 mg/bw-kg groups and positive control compared to the diabetes group from the 3rd week to the end of investigation (*p* < 0.01). Moreover, inflammation was also a key role in the development and progression of diabetic disease, especially the CCL1 is a secreted chemokines by activated T cells that inducing the inflammatory response. In the early observation, the effect of triperpenes on the reducing CCL1 expression was not significantly. However, continuous oral with various concentrion of triterpenes (10 mg/bw-kg, 20 mg/bw-kg and positive control) for 5 weeks, this affect was shown to reduce the serum CCL1 concentration in the 5th week (*p* < 0.01).

**Figure 4:**
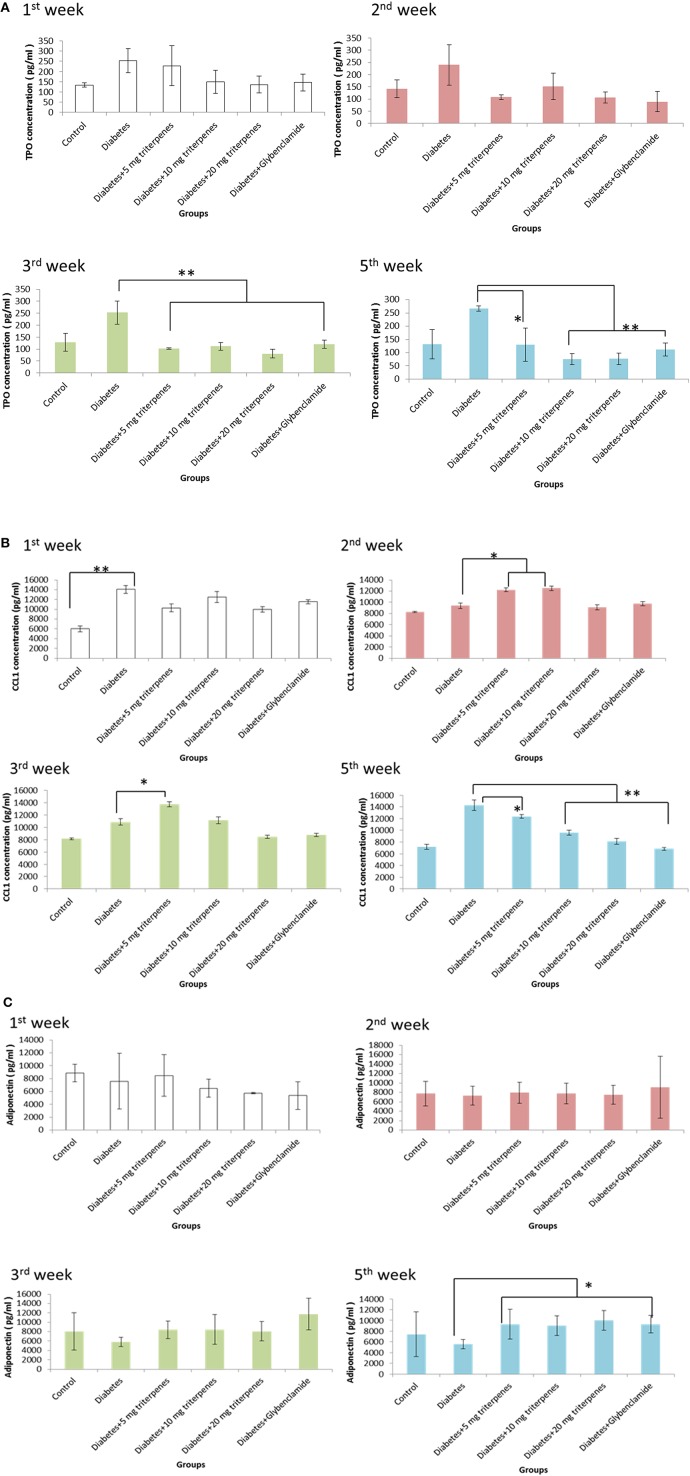
**The TPO concentration was measured in the 1st, 2nd, 3rd, and 5th week as presented in (A)**. The alteration of CCL1 was recorded in the 1st, 2nd, 3rd, and 5th week as presented in **(B)**. The adiponectin was measured 1st, 2nd, 3rd, and 5th week as presented in **(C)**. (*n* = 5, *p*-value less than 0.05 as marked as ^*^ and less than 0.01 as marked as ^**^ was considered to be statistically significant. Results were presented as means ± SEM).

Adiponection induces peripheral insulin sensitivity and inhibition of liver gluconeogenesis, in this investigation of adiponectin observation, it was found that the concentration of adipoectin was not significant exchanged between control and treatment grouped. However, with the time elapsed, the concentration of adiponectin in the treatment groups was significantly induced and expression compared to the diabetes group (*p* < 0.05). We thought that daily oral with triterpenes was supposed to function on the reducing CCL1 concentration and inducing diponectin expression with the elapsed time as presented in Figure 4.

### Diabetes mice wound recovery

**Figure 5:**
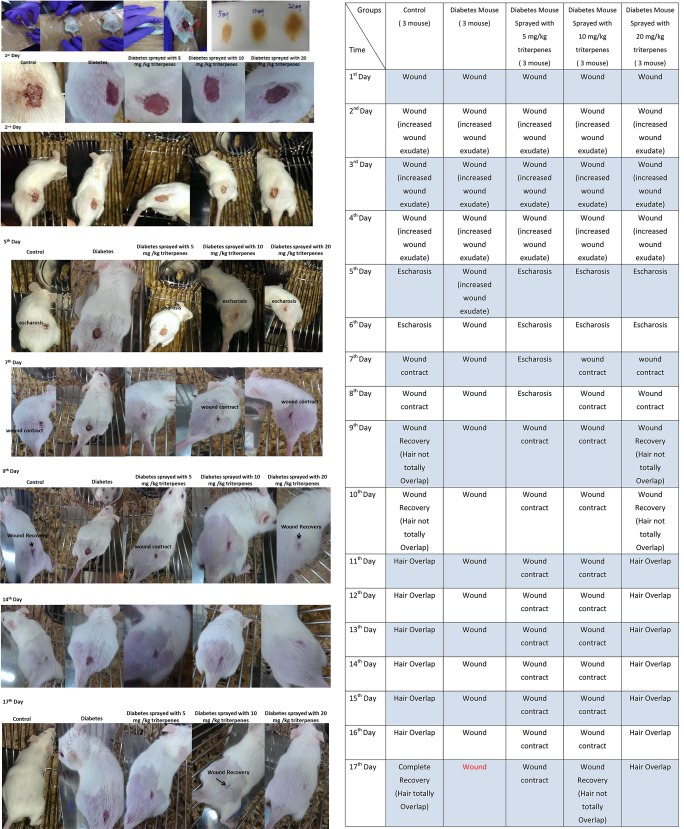
**The wound healing investigation of diabetes mice, the presented observation was indicated to spray with the 20 mg/kg triterpenes with 3M Cavilon^TM^No Sting Barrier Film was exactly able to induce the diabetes mice into wound healing (*n* = 3)**.

The wounded skin recovery assay was observation of area change circumstances. The surgical wound area observation of the five groups was as shown in the Figure 5. The presented as shown in the control group (without diabetes) respectively comparing with the diabetes, sprayed with 5, 10, and 20 mg/kg triterpenes is significantly different in the Days 1–17 after the surgery. In the observation of Day 1 to the Day 5, the presented data has shown that the recovery of the wounded area is markedly in the control and sprayed with 20 mg/kg triterpenes group but not in the diabetes and other treatment groups. In the control group, the recovery process is significantly observed in the Day 5 followed the surgery but the diabetes group has not shown the wound recovery situation in the Day 5. In the Day 7 observation, the wound was initiated to be contacted in the control and 20 mg/kg triterpenes group, other treatment groups was not significant presented with recovery especially the diabetes group. The long-term observation of the wound healing process can be found that the control group completely healing in the Day 17. The 20 mg/kg triterpenes group was not completely wound healing (criteria was presented as hair totally overlapped on the wound) however, the wound exactly has been gradually recovery compared to the diabetes group.

## Discussion

This study evaluated the effects of triterpenes on the regulation of STZ-induced hyperglycaemic diabetes through anti-inflammation response. The diabetic mice were administered daily oral doses of various concentrations of triterpenes, and their which weight gain, volume of drinking water, and liver and spleen weight were recorded. These evaluations revealed more positive regulation of their abnormal metabolism compared with the diabetic mice. In the diabetic mice, the detection of adiponectin production or elevated levels of inflammatory factors such as CCL1 and TPO expression were found to reduce hyperglycaemia and thereby induce an inflammatory response. Moreover, to the best of our knowledge, hyperglycaemia impairs the tissue healing associated with an increased and prolonged inflammatory response. An investigation of the anti-inflammatory response in wound healing as affected by the triterpenes verified the promotion of wound recovery.

Hyperglycaemia associated with diabetes can cause the modification of macromolecules, such as by forming AGE, which can augment the production of proinflammatory cytokines and other inflammatory pathways in vascular endothelial cells. Beyond hyperglycaemia, the diabetic state promotes oxidative stress, mediated by reactive oxygen species and carbonyl groups. Moreover, obesity-induced insulin resistance and diabetes stimulate synthesis of the triglyceride-rich lipoprotein VLDL, which can lower HDL cholesterol by augmenting the transfer from HDL to VLDL via the cholesteryl ester transfer protein. Thus, obesity itself promotes inflammation and potentiates atherogenesis independent of effects on insulin resistance or lipoproteins (Yudkin et al., 1999; Libby et al., 2002). A previous study confirmed that oxidative stress, mitochondrial biogenesis, and aging are all factors in the mitochondrial function that causes insulin resistance (Kim et al., 2008). Another study demonstrated that intracellular levels of ROS rapidly rise, activating various oxidases and peroxidases in response to certain environmental changes (Apel and Hirt, 2004). ROS forms either through energy- or electron-transfer reactions, leading to the formation of singlet oxygen; electron transfer reactions result in the sequential reduction of superoxide, hydrogen peroxide, and hydroxyl radical (Klotz, 2002). This suggests that ROS is a crucial factor in immune cells, which function to trigger cascades of inflammatory activity. In the microenvironment of inflammation related to cellular transdifferentiation, migration, proliferation, survival, and extracellular matrix formation, the growth factors likely to be involved are PDGF, TNF-α and TNF-β, HGF, TGF-β2, epidermal growth factor, fibroblast growth factor, and cytokines such as IL-1, IL-6, IL-8, and IL-10; interferon gamma (INF-γ) is also thought to play a role (Kipanyula et al., 2013; Kisielewski et al., 2013; Morescalchi et al., 2013; Roshani et al., 2014; Przybyt et al., 2015). These factors are clearly involved in maintaining the balance between appropriate fibroblast activation and the fibrosis resulting from their continued activation. Multiple growth factors have been implicated in fibroblast migration and activation, but much attention has recently been focused on the PDGF family of growth factors and their cognate receptors (Nemenoff, 2012a,b). Research has documented that PDGF exerts autocrine and mitogenic effects on keratinocytes to support epidermal proliferation and the stabilization of the epidermal junction during wound closure. In addition, PDGF stimulates vessel maturation through the recruitment and differentiation of pericytes to the immature endothelial channel (Hellberg et al., 2010; Liu et al., 2014; Shimizu et al., 2014; Spiller et al., 2014).

A recent study identified the function of bioactive adiponectin in the physiological homeostasis of glucose and lipid metabolism as well as the elimination of adiponectin, which may be caused by adipocyte enlargement and adipose tissue inflammation (Yamauchi et al., 2001). Adiponectin can suppress the expression of adhesion molecules, such as intracellular adhesion molecules, by inhibiting the TNFα-mediated activation of nuclear factor kappa B in endothelial cells, causing the suppression of monocyte adhesion, thus further inhibiting the proliferation of myelomonocytic lineage cells and suppressing mature macrophage functions, including phagocytic activity and lipopolysaccharide-induced TNFα production (Ouchi et al., 2000; Hu et al., 2013; Matsuda and Shimomura, 2014). A previous study demonstrated that insulinemia and glycaemia, as well as impaired insulin response of glucose oxidation, are independent predictors of high circulating TPO levels (Maury et al., 2010) and that glycaemia, insulinemia, and insulin resistance are contributing factors to TPO levels because hyperglycaemia is positively related to enhanced platelet activation in patients with acute coronary syndrome. This relationship suggests that diabetes may be linked to cardiovascular disease through TPO (Undas et al., 2008).

Evidence shown that expression of CCL1, CXCL9, and CCL21 protein are associated with the infiltrating cells in the inflammatory response (Martin et al., 2007). Downregulation of MCP-1/CCL1 is able to generate the anti-inflammatory mediators such as IL-10 (Börgeson and Godson, 2012). Integration of the recent research and our finding, the triterpenes is capable to reduce the level of the CCL expression in the inflammation response.

*G. lucidum* extract (GE) is able to regulate the differentiation of 3T3-L1 pre-adipocytes and the gene expression of adiponectin (Shimojo et al., 2011). The ability of triterpenes is evidence to inhibit the inflammation in the tumor micron-environment associated with greater reduction of Cox-2 and Twist1 proteins more inhibition of activation of IGF-1R, Stat3, and Src (Cho et al., 2015). By these finding, we speculate that the triterpenes is able to regulate the adiponectin gene expression of adipocyte further to reduce the un-normal metabolism disease, e.g., diabetes.

**Figure 6:**
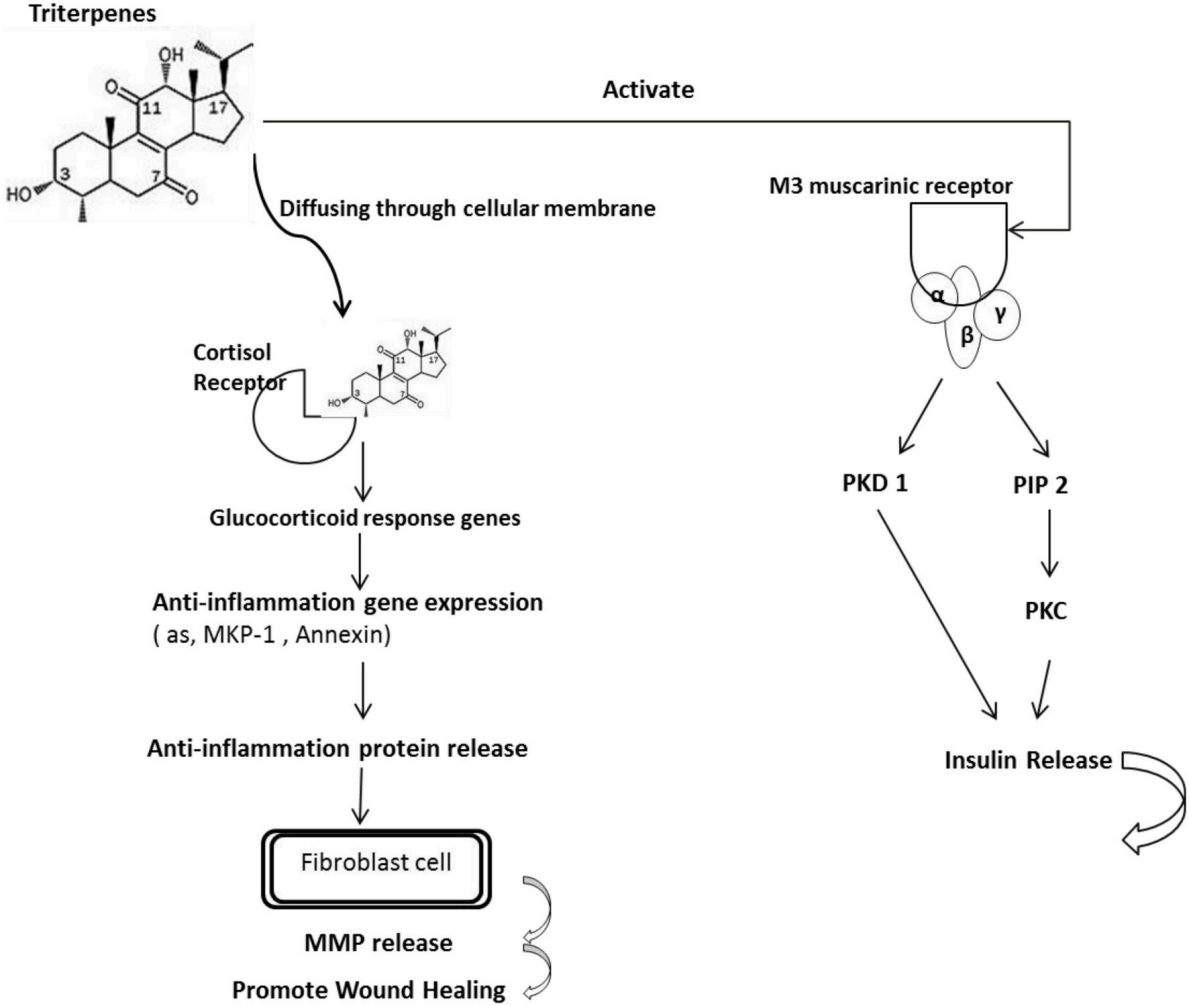
**The proposed mechanism of triterpenes is involved in the diabetes mice with anti-inflammation**. The mechanism of triterpenes was speculated as binding to the glucocorticoid responsive elements (GREs) of target genes to regulate gene expression such as by suppressing the expression of proinflammatory proteins and enhancing the expression of anti-inflammatory proteins. Furthermore, oleanolic acid is a triterpene that can increase insulin secretion by activating muscarinic M3 receptors in pancreatic β-cells through the ACh released from cholinergic nerve terminals. According to these findings, we suggest that the extracted triterpenes could directly permeate the cell to bind with the GRE or indirectly combine with the M3 receptor, resulting in an anti-inflammatory effect and thereby inducing wound healing in the diabetic mice.

The mechanism of triterpenes was speculated as binding to glucocorticoid responsive elements (GRE) of target genes to regulate gene expression, such as suppressing the expression of pro-inflammatory proteins and enhancing the expression of anti-inflammatory proteins (Chen et al., 2011). Further, the oleanolic acid, one of the triterpenes is with the ability to increase insulin secretion *via* an activation of muscarinic M3 receptors in pancreatic β-cells through the released ACh from cholinergic nerve terminals (Hsu et al., 2006; Castellano et al., 2013). Based on these finding, we suggest that the extracted triterpenes may directly bind with the GRE or indirectly combined with the M3 receptor resulting in anti-inflammation response to induce the wound healing as presented as Figure 6.

## Author contributions

SC is the chief of this project. YW is main in the experiment cooperation and idea thinking. Prof Nan is major in the machine cooperation. SC is major in the triterpenes analysis.

### Conflict of interest statement

The authors declare that the research was conducted in the absence of any commercial or financial relationships that could be construed as a potential conflict of interest. The reviewer JJ declared a shared affiliation, though no other collaboration, with the authors the authors YW and SC to the handling Editor, who ensured that the process nevertheless met the standards of a fair and objective review.
