# Supplementary material for: Extracted Triterpenes from Antrodia cinnamomea Reduce the Inflammation to Promote the Wound Healing via the STZ Inducing Hyperglycemia-Diabetes Mice Model
Source: Front Pharmacol. 2016 Jun 13;7:154. doi: 10.3389/fphar.2016.00154 (PMC4904009; doi:10.3389/fphar.2016.00154)

Supplement Data

Detection of methanol extracted materials are including of aflatoxin or not. The detection method was following to the RIDA Quick Aflatoxin (R5024, R-Biopharm AG, Germany) and the detection result was indicated that the extracted materials are not containing with the aflatoxin or less than the 4 ppb.

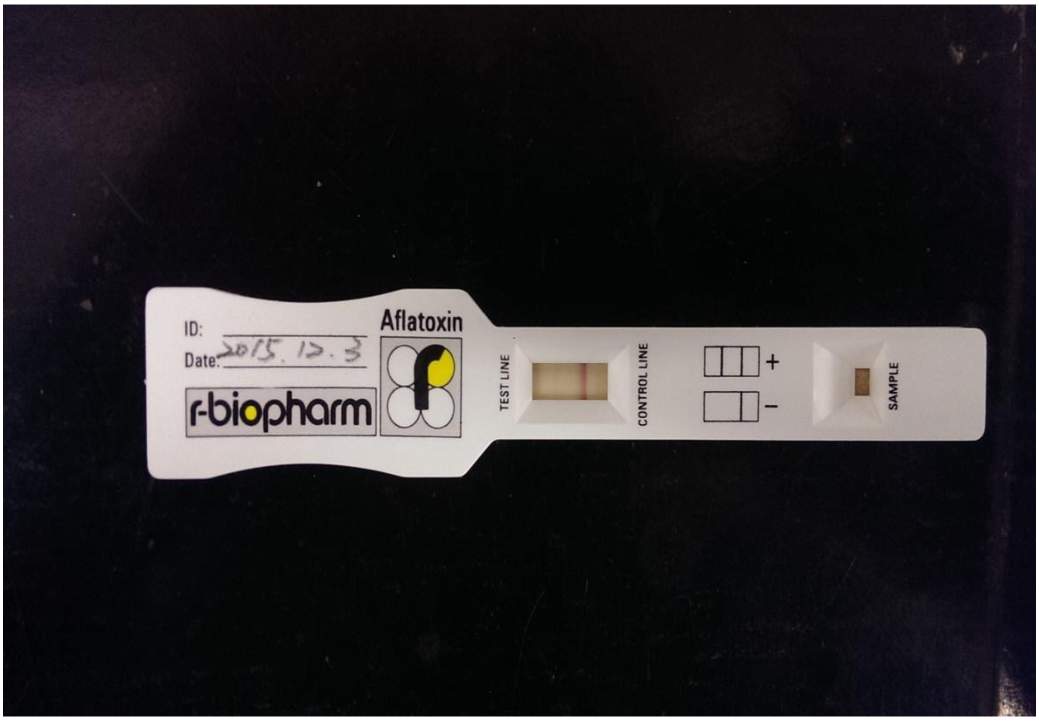

Supplement: Supplementary file 1 [file Presentation1.PDF]
